# Medication reconciliation in clinical practice: a survey of knowledge, attitude, and practices among Egyptian healthcare providers

**DOI:** 10.1186/s12909-025-08285-2

**Published:** 2025-12-19

**Authors:** Aya M. AbdelMagid, Nirmeen A. Sabry, Ahmed Abuelhana, Aaron Courtenay, Amani M. Ali

**Affiliations:** 1 Clinical Pharmacy Department, Faculty of Pharmacy, Cairo University, Kasr El-Aini St., Giza, P.O. Box: 11562, Egypt; 2 Clinical Pharmacy and Pharmacy Practice Department, School of Pharmacy and Pharmaceutical Sciences, Ulster University, Coleraine, UK

**Keywords:** Medication reconciliation, Patient safety, Healthcare providers, Medication error, Pharmacists, Physicians

## Abstract

**Background:**

Patients are at significant risk of harm from unintended medication errors, which remain prevalent and often preventable in healthcare systems, especially in low- and middle-income countries. Medication reconciliation (MedRec) plays a critical role in minimizing these errors by ensuring an accurate and complete medication list throughout patient transitions in the healthcare system.

**Aim:**

This study aimed to assess the current status of the MedRec process in Egypt by exploring the knowledge, attitudes, and practices of physicians and pharmacists as key stakeholders in its implementation.

**Methods:**

This descriptive, cross-sectional study used a structured, validated, self-administered online questionnaire targeting Egyptian physicians and pharmacists. The questionnaire was distributed via snowball sampling through professional networks and social media. Branching logic was used to tailor questions based on respondents’ familiarity with MedRec and hospital-based practices. Descriptive statistics were used to summarize the responses, while Chi-square and Mann–Whitney U tests were used to assess group differences.

**Results:**

Among 272 respondents (182 pharmacists, 90 physicians), representing multiple Egyptian governorates and healthcare sectors (public, private, and military). Among them, 66.9% reported familiarity with MedRec, significantly higher among pharmacists (73.1% vs. 54.4%; *p* = 0.002). Most respondents (70.2%) rated MedRec as “very valuable” for patient safety, and both groups expressed strong responsibility for its core tasks. Among the 136 hospital-based respondents familiar with MedRec, 79.4% reported institutional implementation. Physicians were more likely than pharmacists to ask patients for their current medication lists (93.8% vs. 76.1%; *p* = 0.021). Sources of medication history included patient lists or physician documents (74.3%), family interviews (74.3%), medication boxes from home (59.56%), discharge orders (52.2%), and transfer orders from other facilities (33.8%).

**Conclusion:**

Continuous education and training programs are required to close knowledge gaps, strengthen MedRec practices, and promote a culture of patient safety in Egypt.

**Supplementary Information:**

The online version contains supplementary material available at 10.1186/s12909-025-08285-2.

## Highlights

• Medication reconciliation (MedRec) is widely recognized by healthcare providers as a critical process for enhancing patient safety, preventing medication errors, and ensuring safe transition of care.

• Pharmacists showed greater familiarity with the process, likely due to more educational exposure, whereas physicians were more actively involved in obtaining initial medication histories from patients.

• Both groups demonstrated a strong sense of responsibility and positive attitudes toward the implementation of MedRec across all care transitions, including admission, transfer, and discharge.

• Despite reported institutional adoption, variations in documentation methods and discharge practices highlight the need for standardized training and system-level support.

**Supplementary Information:**

The online version contains supplementary material available at 10.1186/s12909-025-08285-2.

## Introduction

While receiving care within the healthcare system, patients may experience significant harm owing to unintended medical errors. These errors can include unintentional omission, addition, or duplication of medications or incorrect changes in medication dosages. Such errors affect up to 67% of hospitalized patients, with approximately 11% to 59% of these incidents resulting in potential harm [1].

According to the Joint Commission, obtaining and disseminating accurate medication history for patients is one of the crucial National Patient Safety Goals (NPSG) [2]. Medication reconciliation (MedRec) is defined as the process of identifying the most accurate and complete list of all medications a patient is currently using to ensure safe and appropriate medical care [3]. This definition highlights the critical role of MedRec in detecting and reducing prescribing inconsistencies, thereby helping to prevent medication-related problems, prescribing errors, and potential patient harm [2, 4]. This process requires collaboration among different healthcare professionals, with pharmacists and physicians serving as essential partners in ensuring patient safety and delivering optimal care [5].

To fulfil its aim, MedRec should ideally be conducted at every stage of a patient’s transition within the healthcare system, upon admission, during transfers between departments, and at discharge. The ultimate goal is to establish the best possible medication history (BPMH) at admission and the best possible medication discharge plan (BPMDP) at discharge [6]. In addition, MedRec can be performed by community pharmacists in outpatient settings to help ensure patient safety across all areas of care [7].

The World Health Organization (WHO) has issued a standard operating protocol (SOP) for implementing MedRec, which outlines four essential steps in the process. These steps include gathering and structuring the BPMH, verifying its accuracy, and reconciling it with prescribed medications. Finally, accurate medication information should be communicated to the next care providers, whether that is the patient, their caregiver, or the healthcare professional involved in the subsequent stage of care [8].

Medication safety remains a significant challenge in many low- and middle-income countries (LMICs), including those in Northern and Eastern Africa. Limited resources, poor communication, low health literacy, and fragmented healthcare systems often contribute to preventable medication errors and patient harm [9, 10]. In a study conducted in an Egyptian intensive care unit (ICU), it was reported that up to 97% of patients had at least one medication-related problem. The most common types of errors were dosing errors, therapeutic duplication, and the use of unnecessary medications [11].

Importantly, this process requires dedicated effort and considerable time from healthcare professionals, which can present a significant barrier to implementation in LMICs [3]. Additional barriers include poor communication, low health literacy, and information loss [12].

There is a scarcity of studies exploring medication safety in LMICs, including Egypt [10]. The limited available literature has mainly evaluated current clinical pharmacy practices and their economic impact, such as studies on MedRec accuracy in emergency departments and the economic value of clinical pharmacy interventions [13–15]. However, understanding healthcare professionals’ knowledge, attitudes, and practices (KAP) is a crucial first step to identifying gaps, informing targeted interventions, and guiding future practices and educational programs to address these gaps. Therefore, in the present study, we aimed to assess the status of MedRec in Egypt by examining the KAP of healthcare professionals. We focused on the perspectives of physicians and pharmacists, as they are key stakeholders in MedRec implementation.

## Methods

### Study design and population

This descriptive, cross-sectional, questionnaire-based study was conducted online to assess healthcare providers’ KAP regarding the MedRec process in Egypt. This study targeted pharmacists and physicians working in various healthcare settings, including public, private, and military institutions. The study protocol was reviewed and approved by the Ethics Committee of the Faculty of Pharmacy, Cairo University (CL3929). This cross-sectional study was reported in accordance with the STROBE (Strengthening the Reporting of Observational Studies in Epidemiology) guidelines [16] to ensure methodological transparency and completeness.

### Questionnaire development

This study utilized a validated self-administered online questionnaire adapted from a survey from a previous study in English [17]. English was retained as the questionnaire language as it is the primary language of medical and pharmaceutical education and professional communication among healthcare providers in Egypt. Therefore, all respondents were expected to possess adequate English proficiency to complete the questionnaire. The questionnaire consisted of 33 questions in four sections to be completed within 15 min. These questions covered demographics and professional background (11 questions), knowledge and educational exposure to MedRec (3 questions), attitudes and perceived responsibilities (8 questions), and practices related to MedRec process implementation (11 questions). The tool included binary (yes/no), multiple-choice, and 5-point Likert scale questions. All responses, including “I don’t know,” were coded into distinct categories. The questionnaire was reviewed for face and content validity prior to distribution by ten experts in the field, including clinical pharmacists, staff members of the clinical pharmacy department at Cairo University, and physicians. They provided feedback on the clarity and relevance of the questions and ensured that the items were appropriate and covered the key aspects of the KAP of the MedRec process. Minor adjustments were made to wording and layout based on their suggestions to improve the clarity of the questions without any change to the core of the content. The questionnaire was open to all pharmacists and physician respondents. As suggested by the reviewers, a branching structure-guided survey flow was adopted. Respondents who reported working in a hospital setting and were familiar with the MedRec process completed all four sections, including practice-related items. Those unfamiliar with MedRec or not working in hospitals completed only the demographic, knowledge, and attitude sections. The final questionnaire was created using a Google Forms^®^ link.

### Sampling and recruitment

The Google Forms^®^ link of the questionnaire was disseminated electronically via social media platforms such as Facebook^®^, WhatsApp^®^, and LinkedIn^®^, which serve as common channels of professional communication among Egyptian healthcare providers. This approach offered a practical and cost-effective means to reach a geographically dispersed target population in the absence of a centralized contact registry. To maximize outreach, a snowball sampling technique was employed, whereby participants were encouraged to forward the survey link to colleagues within their professional networks. This method was employed because no comprehensive national registry of healthcare providers directly involved in the MedRec process was available, and the target population was widely distributed across different healthcare sectors and governorates. It also enabled the inclusion of participants from diverse healthcare settings beyond the researchers’ immediate reach. Data was collected anonymously using an online platform from May 1, 2025, to June 22, 2025. No personal identifiers such as names, emails, or IP addresses were collected to ensure participants’ confidentiality. The cover page preceding the questionnaire outlined the study purpose and served as an agreement to participate in the study. Completion of the questionnaire was voluntary, and anonymity was assured during data analysis. Participants were informed that their responses would remain confidential and that they could withdraw at any time without penalty. Participants were not involved in the development of research questions, outcome measures, or study design. They did not participate in the interpretation or writing of the results.

### Sample size calculation

Currently, no standardized method for sample size calculation is specific to KAP studies. Therefore, we adopted a commonly used psychometric approach that recommends recruiting 5 to 10 participants per questionnaire item [18]. With 33 items in our KAP questionnaire, a minimum of 165 participants was considered sufficient. Additionally, to assess the adequacy of the final sample size, a margin of error calculation was performed using an online calculator (https://www.calculator.net/sample-size-calculator.html). A post hoc power analysis was conducted using G*Power (version 3.1.9.2) to confirm the adequacy of the final sample for detecting group differences under the chosen significance level.

### Statistical analysis

Statistical analyses were performed using Statistical Package for Social Sciences (SPSS^®^) for Windows Version 25.0 (IBM Corp., Armonk, NY). Descriptive statistics (frequency and percentage) were used to summarize participant characteristics and responses. Group comparisons between pharmacists and physicians were conducted using the chi-square test or Fisher’s exact test for categorical variables and the Mann-Whitney U test for ordinal data which did not meet the assumptions of normality required for parametric tests. Following a significant chi-square test (*p* < 0.05), adjusted standardized residuals were examined to identify individual cells contributing to the overall association. Values exceeding ± 1.96 were considered statistically significant at *p* < 0.05. Binary logistic regression was employed to identify predictors of familiarity with the MedRec process, with results presented as adjusted odds ratios (ORs) and 95% confidence intervals (CIs). No imputation was required, as all questionnaire items were mandatory in the online Google form, ensuring complete responses for both descriptive and regression analyses. The option “I don’t know” was treated as a valid response category and analyzed descriptively. Statistical significance was set at p-value < 0.05. Figures were generated using Microsoft Excel^®^.

## Results

### Demographic characteristics

A total of 272 healthcare providers participated in the study, including 182 pharmacists (182/272, 66.9%) and 90 physicians (90/272, 33.1%). Approximately half of the respondents (136/272) worked in a hospital and were familiar with the MedRec process; therefore, they proceeded to answer the practice section. The remaining respondents (136/272) answered only the knowledge and attitude sections, as they were either unfamiliar with the MedRec process or did not work in a hospital setting. The demographic characteristics of all respondents, stratified by healthcare profession, are summarized in Table 1. It has been shown that most respondents were female (194/272, 71.3%), with a significantly higher proportion of females among pharmacists compared to physicians (77.5% (141/182) vs. 58.9% (53/90), *p* = 0.001). Participants aged 31–40 years were the most represented group (99/272, 36.4%), with pharmacists generally younger than physicians (*p* < 0.001). Compared with physicians, pharmacists were significantly more likely to hold a bachelor’s degree (37.4% (68/182) vs. 17.8% (16/90)), postgraduate diploma (12.1% (22/182) vs. 1.1% (1/90)), or PhD (19.2% (35/182) vs. 2.2% (2/90)). In contrast, a significantly higher proportion of physicians held a Doctor of Medicine (MD) degree (37.8% (34/90) vs. 0% (0/182)) (all *p* < 0.001). Approximately half of the respondents had more than 10 years of professional experience (134/272, 49.3%), with no significant difference between groups. Compared to pharmacists, physicians were more likely to work more than 8 h per day (82.2% (74/90) vs. 62.1% (113/182), *p* = 0.001) and handle more patients daily (*p* < 0.001). Additionally, a significantly higher proportion of physicians worked in hospital settings than pharmacists (96.7% (87/90) vs. 53.8% (98/182); *p* < 0.001). There was no statistically significant difference in the type of healthcare institution between groups (*p* = 0.259). The demographic characteristics of respondents who completed all questionnaire sections versus those who did not are summarized in Supplementary Tables S1 and S2.

**Table 1:** Demographic data of respondents

Parameter	All (*N* = 272)	Pharmacist *N* = 182	Physician *N* = 90	*P* value
Gender, Female	194 (71.3)	141 (77.5)	53 (58.9)	0.001*
Age (Years)				
23–30 years	90 (33.1)	68 (37.4)	22 (24.4) ^a^	< 0.001*
31–40 years	99 (36.4)	80 (44)	19 (21.1) ^a^	
41–50 years	59 (21.7)	32 (17.6)	27 (30) ^a^	
51–60 years	19 (7)	1 (0.5)	18 (20) ^a^	
61 or above	5 (1.8)	1 (0.5)	4 (4.4) ^a^	
Highest Academic Degree				
Bachelor’s degree	84 (30.9)	68 (37.4)	16 (17.8) ^a^	< 0.001*
Master’s degree	80 (29.4)	48 (26.4)	32 (35.6)	
Post-graduate diploma	23 (8.5)	22 (12.1)	1 (1.1) ^a^	
PhD Degree	37 (13.6)	35 (19.2)	2 (2.2) ^a^	
MD Degree	34 (12.5)	0 (0)	34 (37.8) ^a^	
Other	14 (5.1)	9 (4.9)	5 (5.6)	
Years of Experience				
< 2 years	19 (7)	12 (6.6)	7 (7.8)	0.113
2–5 years	66 (24.3)	46 (25.3)	20 (22.2)	
6–10 years	53 (19.5)	42 (23.1)	11 (12.2)	
> 10 years	134 (49.3)	82 (45.1)	52 (57.8)	
Average Working Hours per Day				
< 8 h	85 (31.3)	69 (37.9)	16 (17.8) ^a^	0.001*
≥ 8 h	187 (68.8)	113 (62.1)	74 (82.2) ^a^	
Number of Patients Dealt with During a Typical Working Day				
None	77 (28.3)	75 (41.2)	2 (2.2) ^a^	< 0.001*
1–10	55 (20.2)	36 (19.8)	19 (21.1)	
11–20	70 (25.7)	31 (17)	39 (43.3) ^a^	
21–30	23 (8.5)	14 (7.7)	9 (10)	
> 30	47 (17.3)	26 (14.3)	21 (23.3)	
Working in a Hospital	87 (31.98)	98 (53.8)	87 (96.7)	< 0.001*
Healthcare Institution Type				
Public	126 (46.3)	62/98 (63.3)	64/87 (73.6)	0.259
Private	46 (16.9)	27/98 (27.6)	19/87 (21.8)	
Military	13 (4.8)	9/98 (9.2)	4/87 (4.6)	

*Abbreviations: MD* Doctor of Medicine, *PhD* Doctor of Philosophy

Categorical data as Number (Percentages)

*:Level of significance *P* < 0.05, Chi Square (two -sided)

^a﻿^Significant values of adjusted standardized residuals (absolute value > ± 1.96 indicate statistical significance at *p* < 0.05)

### Knowledge regarding MedRec

#### Familiarity and educational exposure

Familiarity with the MedRec process was reported by 66.9% (182/272) of respondents, with pharmacists significantly more likely than physicians to be familiar with the process (73.1% (133/182) vs. 54.4% (49/90), *p =* 0.002). Likewise, pharmacists were more likely than physicians to report receiving MedRec-related education during university training (44.5% (81/182) vs. 22.2% (20/90), *p =* 0.001). However, formal workplace training on MedRec roles did not differ significantly between the groups (*p =* 0.201). Familiarity with MedRec and educational exposure among pharmacists and physicians are presented in Table 2.

**Table 2:** Familiarity with Medrec and educational exposure among pharmacists and physicians

Question	Response	All *N* = 272	Pharmacist *N* = 182	Physician *N* = 90	*P*-value
Are you familiar with the term MedRec?	Yes	182 (66.9)	133 (73.1)	49 (54.4)	0.002*
Receipt of education or training during university degree on MedRec	Yes	101 (37.1)	81 (44.5)	20 (22.2) ^a^	0.001*
	No	152 (55.9)	87 (47.8)	65 (72.2) ^a^	
	I don’t know	19 (7)	14 (7.7)	5 (5.5)	
Attendance of formal education or training at work on your role in MedRec	Yes	94 (34.6)	68 (37.4)	26 (28.9)	0.201
	No	175 (64.3)	113 (62.1)	62 (68.9)	
	I don’t know	3 (1.1)	1 (0.5)	2 (2.2)	

*Abbreviations: MedRec* Medication Reconciliation

Categorical data as Number (Percentages)

*:Level of significance *P* < 0.05, Chi Square (two -sided)

^a^Significant values of adjusted standardized residuals (absolute value > ± 1.96 indicate statistical significance at *p* < 0.05)

#### Determinants of MedRec familiarity among healthcare providers

In the multivariable logistic regression model, pharmacists had higher odds of reporting MedRec familiarity (OR = 6.03, 95% CI: 2.81–12.96, *p <* 0.001), and prior university training or education was a significant predictor (OR = 3.05, 95% CI: 1.03–9.05, *p =* 0.045). In contrast, respondents who were not currently working or had never worked in a hospital setting were significantly less likely to report familiarity (OR = 0.14, 95% CI: 0.07–0.30, *p <* 0.001) (Table 3). Other demographic and professional characteristics, including sex, age, years of experience, working hours, number of patients dealt with, academic degree, and workplace training, were not significantly associated (*p >* 0.05).

**Table 3:** Adjusted odds ratios for predictors of familiarity with the MedRec process among healthcare providers

Variable	OR (Exp(B))	95% CI	*p*-value
Healthcare profession (Pharmacist vs. Physician)	6.03	[2.81–12.96]	< 0.001
Received MedRec education during university – Yes vs. No	3.05	[1.03–9.05]	0.045
Currently working or ever worked in a hospital Yes vs. No	0.14	[0.07–0.30]	< 0.001

*Abbreviations: CI* Confidence Interval, *MedRec* Medication Reconciliation, *OR* Odds Ratio, *vs*. Versus

### Attitude towards MedRec

#### Perceived responsibilities in the MedRec process

**Fig. 1:**
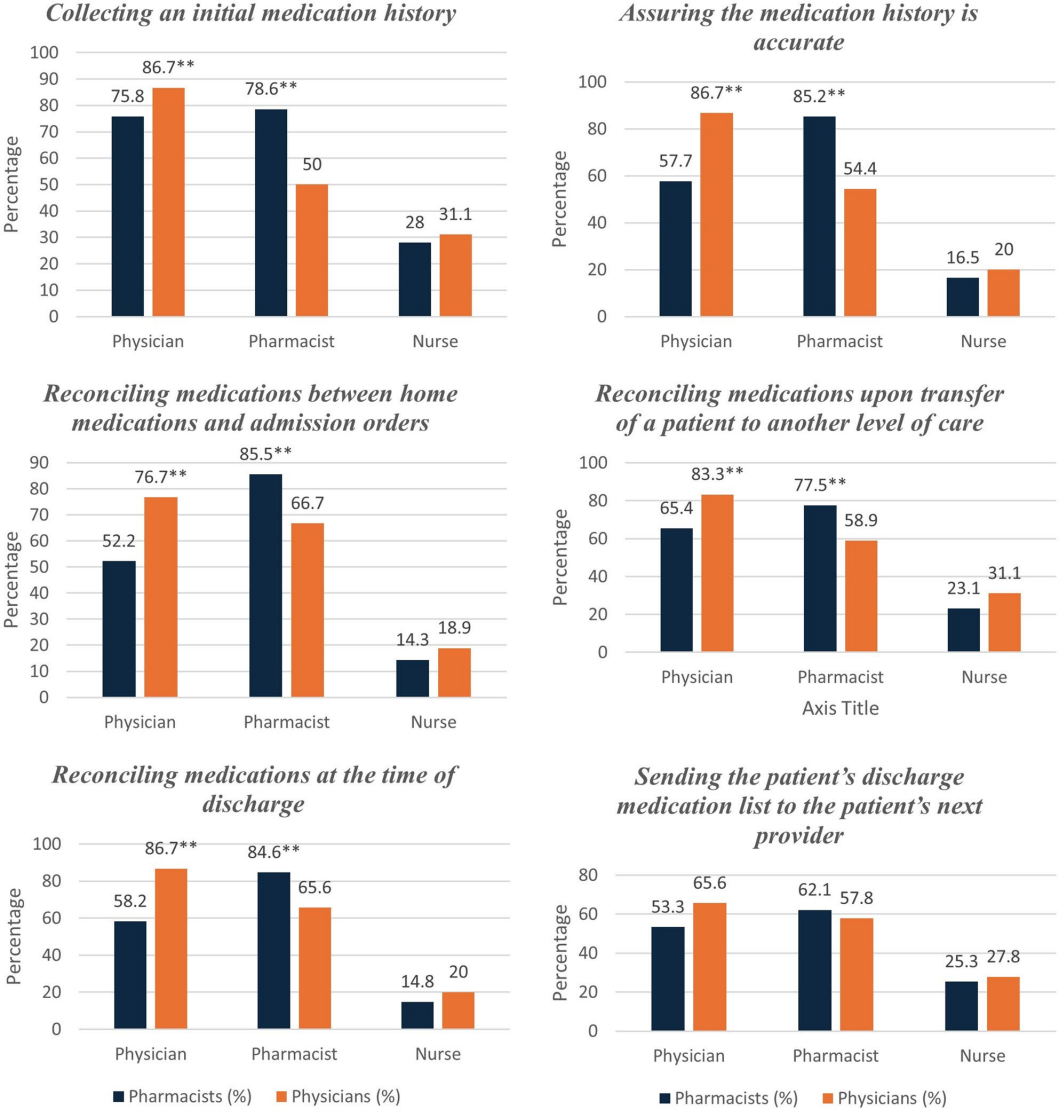
Perceived responsibility for MedRec tasks among pharmacists and physicians (**: level of significance *P* < 0.05, Chi Square (two -sided))

Regarding perceived responsibilities, both groups demonstrated a strong sense of responsibility for MedRec, including collecting initial medication histories, assuring medication history accuracy, and reconciling medications during various transitions of care, including admission, transfer, and discharge, without a significant difference between pharmacists’ and physicians’ responses, as shown in Fig. 1.

#### Overall attitudes and perceived value of MedRec

**Fig. 2:**
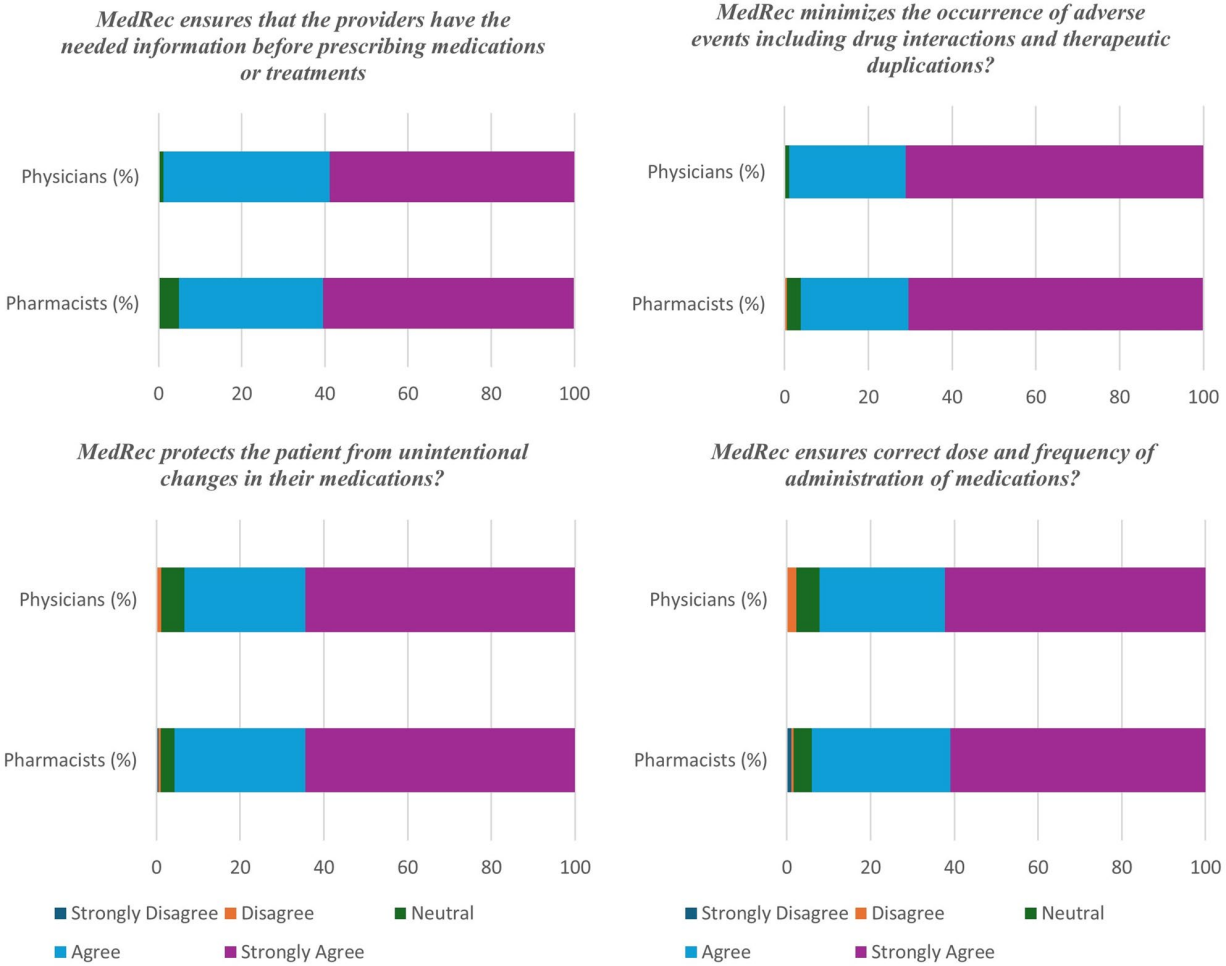
Attitudes of pharmacists and physicians toward the impact of MedRec on patient safety

**Fig. 3:**
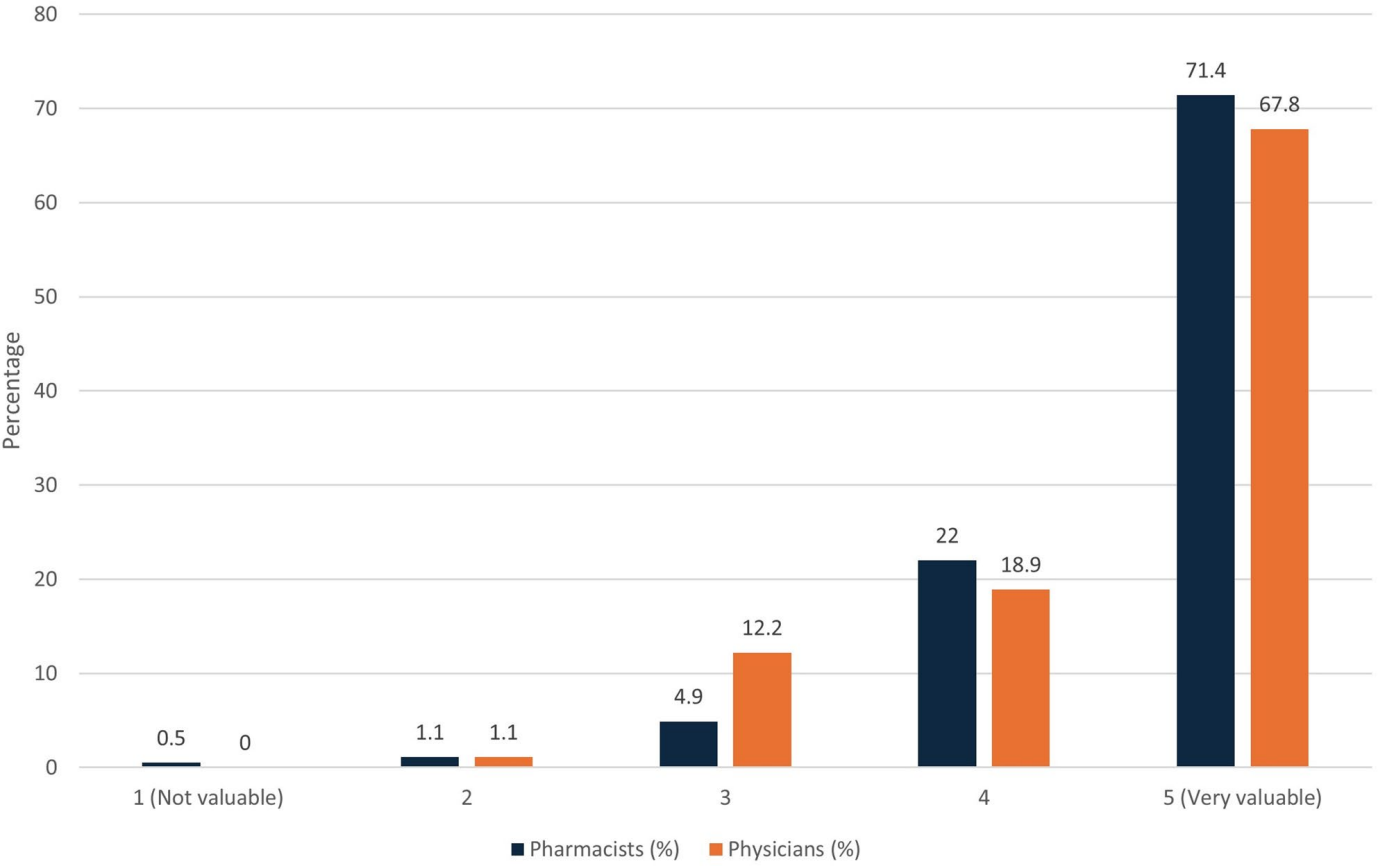
Perception of the overall value of the Medrec process to patient safety

Attitude-related items showed generally positive attitudes that were not significantly different between the two groups. Most respondents agreed or strongly agreed that MedRec ensures necessary information before prescribing (262/272, 96.3%), minimizes adverse events (264/272, 97.1%), prevents unintentional medication changes (258/272, 94.8%), and ensures accurate dosing and frequency (254/272, 93.4%) as shown in Fig. 2. The overall perceived value of MedRec was rated as very valuable (score = 5) by 70.2% (191/272) of participants (Fig. 3).

### Practices of MedRec

#### Implementation of MedRec

**Fig. 4:**
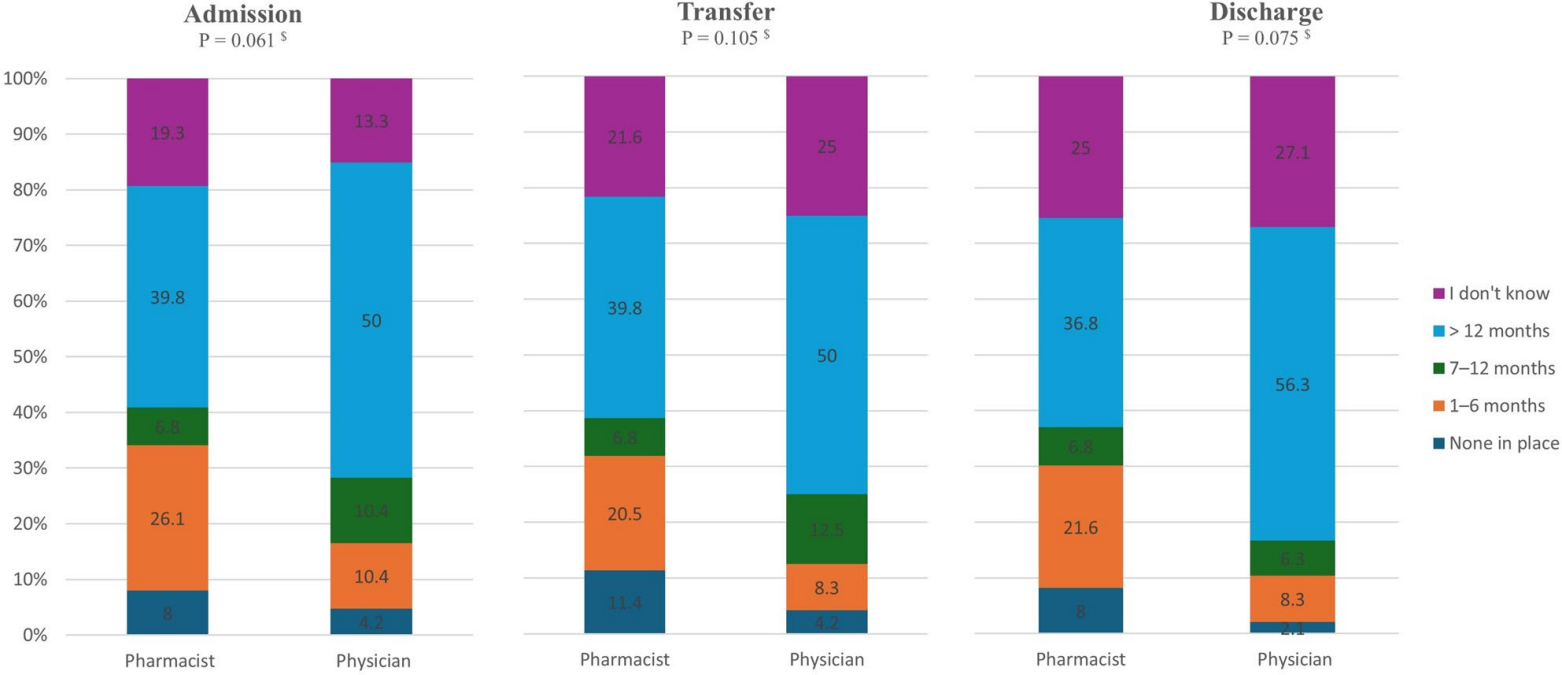
Proportional responses from pharmacists and physicians on how long the Medrec process has been established at admission, transfer, and discharge. ($: independent samples Mann-Whitney U test at *P* < 0.05)

Among the 136 respondents to the practice section, 79.4% (108/136) reported that their workplace implemented MedRec. A notable proportion of physicians (35/48, 72.9%) and pharmacists (52/88, 59.1%) reported personally conducting the MedRec process. Although the difference between the two groups was not statistically significant, the higher rate among physicians may reflect their central roles in admission, transfer, and discharge procedures. Participants were asked to report how long the MedRec process had been established in their department for admission, interdepartmental transfer, and discharge to assess the maturity and continuity of MedRec implementation across different transitions of care. Their responses varied, with over 40% reporting that it had been implemented for more than 12 months (Fig. 4). No significant difference was observed between pharmacists and physicians, and a notable proportion of them responded with ‘I don’t know’ (*P >* 0.05). However, pharmacists were significantly less likely than physicians to routinely ask patients for their current medication list upon arrival (76.1% (67/88) vs. 93.8% (45/48), *p* = 0.021).

#### Medication history collection and documentation practices

Sources used to obtain medication histories included patient medication lists or primary care physician documents (101/136, 74.3%), family interviews (101/136, 74.3%), medication boxes obtained from home (81/136, 59.56%), discharge orders from recent hospitalization (71/136, 52.2%), and transfer orders from other facilities (46/136, 33.8%). The majority in both professions (72.1%) reported interviewing both the patient and their family/carer to obtain the BPMH. Regarding documentation, pharmacists and physicians primarily used paper charts (60.2% (53/88) and 41.7% (20/48), respectively, *p* = 0.003). Approximately half of the institutions provided handwritten current medication lists to patients upon discharge (69/136, 50.7%), and 26.5% (36/136) used computer-generated lists. Most of these lists included medication names (126/136, 92.6%), doses (122/136, 89.7%), routes (117/136, 86%), frequencies (120/136, 88.2%), and durations (112/136, 82.4%), with no significant intergroup differences. The MedRec practices of pharmacists and physicians are summarized in Table 4.

**Table 4:** MedRec practices among pharmacists and physicians

Response	All (*N* = 136)	Pharmacist *N* = 88	Physician *N* = 48	*P*-value		
Does your workplace apply the MedRec process?						
Yes	108 (79.4)	67 (76.1)	41 (85.4)	0.230		
No	21 (15.4)	17 (19.23)	4 (8.3)			
I don’t know	7 (5.1)	4 (4.5)	3 (3.3)			
On average, how many times do you perform MedRec per day?						
Not part of my responsibilities	49 (36)	36 (40.9)	13 (27.1)	0.566^£^		
1–2 times	32 (23.5)	16 (18.2)	16 (33.3)			
3–6 times	27 (19.9)	17 (19.3)	10 (20.8)			
7–10 times	12 (8.8)	7 (8)	5 (10.4)			
> 10 times	16 (11.8)	12 (13.6)	4 (8.3)			
Do you routinely ask patients for a current list of medications when they arrive at your service?						
Yes	112 (82.4)	67 (76.1)	45 (93.8) ^a^	0.021*		
No	7 (5.1)	5 (5.7)	2 (4.2)			
Not Applicable	17 (12.5)	16 (18.2)	1 (2.1) ^a^			
What are the sources you usually use to obtain a list of the current medications to the patient?						
Patient medication list or 1^ry^ care physician documents	101 (74.3)	64/88 (72.2)	37/48 (77.1)	0.579		
Patient family member carer interview	101 (74.3)	68/88 (77.3)	33/48 (68.8)	0.277		
Medication boxes obtained from home	81 (59.56)	55/88 (62.5)	26/48 (54.2)	0.344		
Discharge medication orders from recent hospitalizations	71 (52.2)	44/88 (50)	27/48 (56.3)	0.486		
Transfer orders from other facilities	46 (33.8)	32/88 (36.4)	14/48 (29.2)	0.397		
Do you routinely interview the patient carer or family member during the MedRec process for creation of the BPMH?						
No	5 (3.7)	5 (5.7)	0 (0)	0.106		
Yes, with a family member/carer only	9 (6.6)	7 (8)	2 (4.2)			
Yes, with the patient only	13 (9.6)	5 (5.7)	8 (16.7)			
Yes, with both the patient and a family member/carer	98 (72.1)	63 (71.6)	35 (72.9)			
Not applicable	11 (8.1)	8 (9.1)	3 (6.3)			
Type of form in which the MedRec process is documented						
Not documented	4 (2.9)	1 (1.1)	3 (6.3)	0.003*		
Computer chart	20 (14.7)	6 (6.8)	14 (29.2) ^a^			
Paper chart	73 (53.7)	53 (60.2)	20 (41.7) ^a^			
Combination of both	29 (21.3)	21 (23.9)	8 (16.7)			
I don’t know	10 (7.4)	7 (8)	3 (6.3)			
Does your institution provide a list of current medications to patients when they are discharged or leave the hospital pharmacy?						
No	12 (8.8)	11 (12.5)	1 (2.1)	0.083		
Yes, handwritten, only if patient asked	4 (2.9)	4 (4.5)	0 (0)			
Yes, computer generated, only if patient asked	2 (1.5)	1 (1.1)	1 (2.1)			
Yes, handwritten	69 (50.7)	45 (51.1)	24 (50)			
Yes, computer generated	36 (26.5)	18 (20.5)	18 (37.5)			
I don’t know	13 (9.6)	9 (10.2)	4 (8.3)			
For each medication, does the generated medication list provided to patients include:						
Name	126 (92.6)	82/88 (93.2)	44/48 (91.7)	0.101		
Dose	122 (89.7)	80/88 (90.9)	42/48 (87.5)	0.064		
Route of administration	117 (86)	73/88 (83)	44/48 (91.7)	0.221		
Frequency of administration	120 (88.2)	78/88 (88.6)	42/48 (87.5)	0.903		
Duration of treatment	112 (82.4)	69/88 (78.4)	43/48 (89.6)	0.241		

*Abbreviations: MedRec* Medication Reconciliation

Categorical data as Number (Percentages)

*: Level of significance *P* < 0.05, Chi Square (two -sided)

^£^: Independent samples Mann-Whitney U test at *P* < 0.05

^a﻿^Significant values of adjusted standardized residuals (absolute value > ± 1.96 indicate statistical significance at *p* < 0.05)

### Post-hoc assessment of sample adequacy, power, and reliability

Post-hoc evaluation of the final sample of 272 respondents revealed a margin of error of 5.94% at a 95% confidence level, confirming acceptable precision for population proportion estimates. In addition, a post hoc power analysis using G*Power (v3.1.9.2) for a two-tailed Fisher’s exact test comparing proportions (p₁ = 0.731 vs. p₂ = 0.544, α = 0.05) with a 2:1 allocation (n₁ = 182, n₂ = 90) yielded a power of 85.4%, indicating adequate statistical power to detect the observed difference in MedRec familiarity between the two professional groups.

Also, the internal consistency reliability of the adapted questionnaire was evaluated using Cronbach’s alpha after data collection. The attitude domain (five Likert-scale items) demonstrated good reliability (α = 0.8), while the practice domain (11 items) showed acceptable reliability (α = 0.72). The knowledge domain comprised factual, dichotomous items assessing familiarity, awareness, and training on the MedRec process; therefore, internal consistency testing was not applicable. Overall, these results confirm the internal coherence of the attitudinal and behavioral sections of the questionnaire and support the reliability of the instrument in assessing healthcare providers’ knowledge, attitudes, and practices toward MedRec.

## Discussion

In this cross-sectional study, we examined, for the first time, the KAP of pharmacists and physicians, who represent the core professionals responsible for delivering effective MedRec services in Egypt. Our findings revealed that pharmacists demonstrated higher literacy regarding the MedRec process, likely due to greater exposure to related content during their formal education or through targeted training sessions in the workplace. Notably, the study also emphasized that both pharmacists and physicians view themselves as chiefly responsible for carrying out MedRec at every stage of patient transition in the healthcare system, including collecting and verifying patient’s medication history.

In this survey, the majority of respondents were pharmacists (*N* = 182), while physicians provided only 90 responses. This can be attributed to the fact that, as indicated by some survey questions, physicians tend to work longer hours daily and serve a larger number of patients (Table 1).

Regional and international studies support our findings regarding pharmacists’ relatively higher familiarity with MedRec. For example, a Kuwaiti study across six hospitals similarly reported that pharmacists were more familiar with MedRec than physicians, primarily due to greater exposure during academic training. However, physicians in Kuwait also reported higher rates of workplace training (43.1%) than those in our study (28.9%), underscoring the impact of structured hospital-based programs [17]. This observation is further supported by multiple interventional studies that have demonstrated significant improvements in healthcare providers’ KAP following structured educational programs [19, 20]. Moreover, a non-randomized controlled trial conducted in Nigeria showed that educational interventions on MedRec not only reduced medication discrepancies and the incidence of drug therapy problems but also enhanced patient engagement in the process, for example, by encouraging patients to bring their medication packs to the hospital, thereby facilitating more accurate BPMH reporting [21]. Consistent with our findings, a national survey conducted in the United Arab Emirates showed that prior education or training on MedRec was the main determinant of participants’ knowledge of the process [22].

It is worth mentioning that the Kuwaiti study reported higher overall familiarity with MedRec, as well as greater educational and training support for healthcare professionals, compared to our study findings. According to the World Bank classification, Kuwait is considered a high-income country, which may help explain the wider availability of dedicated MedRec training programs in the workplace, along with stronger institutional support and motivation for healthcare professionals to provide comprehensive MedRec services [23].

In our study, both pharmacists and physicians perceived themselves as primarily responsible for executing the MedRec process, consistent with findings from Kuwait [17]. Similarly, a 2015 Omani study reported comparable perceptions, except that pharmacists assigned physicians greater responsibility for forwarding discharge medication lists, while both groups viewed themselves as equally responsible for admission interviews [24]. A Brazilian multicenter study also found that 66.7% of pharmacists considered physicians mainly responsible for documenting MedRec at discharge [25]. Notably, pharmacists were underrepresented in both the Omani and Brazilian studies, and their reported involvement in MedRec activities was limited, reflecting variability in role perception and practice across settings.

Responsibility for the various steps involved in MedRec remains an area of considerable debate and varies widely across healthcare settings. While most published guidance recognizes MedRec as a multidisciplinary process shaped by institutional policies and national guidelines, few clearly delineate the specific roles and responsibilities of each member of the healthcare team. Nonetheless, there is general agreement that all professionals involved should be adequately familiar with and trained in the procedure to ensure its effectiveness. Notably, pharmacy-focused organizations such as the International Pharmaceutical Federation (FIP) and the American Society of Health-System Pharmacists (ASHP) emphasize that pharmacists are key players and should assume primary responsibility for implementing the process, as well as for training other healthcare professionals [7, 26]. In addition, the WHO highlights that pharmacists should ideally be involved, at a minimum, in the initial steps of gathering, verifying, and reconciling the BPMH [8].

In our study, both pharmacists and physicians perceived themselves as the primary professionals responsible for executing the MedRec process at all transition points, including collecting and verifying medication histories. This mirrors the divergence highlighted in international literature. For example, Kirby et al. found that while clinicians generally agree that physicians are responsible at admission and discharge, there is substantial disagreement regarding who should obtain medication histories or provide discharge counseling [27]. Similarly, Mueller et al.’s systematic review showed wide variability in how responsibilities are assigned across hospitals, with roles shared among nurses, physicians, and pharmacists depending on institutional policies [28]. Our findings reflect this global ambiguity, underscoring the need for clear role delineation within Egyptian healthcare institutions through national or facility-level guidelines to ensure consistent and effective MedRec practices.

Another important finding, consistent with the results of the Kuwaiti study, is that pharmacists were significantly less likely than physicians to ask patients for a current list of medications upon arrival at the care site (76.1% vs. 93.8%, *p* = 0.021). This discrepancy may be attributed to the fact that patients are more likely to meet physicians during scheduled outpatient visits or at the time of admission, before any interaction with pharmacists [29]. This gap highlights opportunities for targeted interventions to enhance pharmacists’ involvement early in the patient journey, such as integrating them into admission workflows or outpatient triage points. Furthermore, these differences underscore the need for profession-specific training—for physicians, structured workplace programs to strengthen their familiarity with the MedRec process; and for pharmacists, capacity-building focused on patient interviewing and documentation skills. At the policy level, hospital guidelines should clearly define responsibilities and promote collaborative, team-based MedRec practices to ensure accuracy and consistency across care transitions.

An interesting finding was that the use of paper charts was higher than computer charts for documenting the MedRec process (Table 4). This likely reflects a broader reliance on manual documentation methods, which may stem from limited access to electronic health records in many practice settings. In Egyptian hospitals, digitization remains uneven across institutions, and healthcare professionals often face restricted or fragmented access to electronic systems [30]. Although differences in reported workplace implementation of MedRec were not statistically significant, the observed trends align with these documentation patterns and underscore broader system-level gaps in digitization and standardization that warrant attention.

The current study has several limitations. First, the relatively small sample size, likely influenced by the online survey format and the busy schedules of participating healthcare practitioners, may have resulted in wide confidence intervals and reduced the precision of some estimates. Second, snowball sampling, as a non-probability sampling method, may have introduced selection bias and limited the representativeness of the sample, favoring individuals with higher educational level, stronger professional networks or a greater interest in the topic. Third, internal consistency reliability was evaluated only after data collection and was limited to attitude and practice domains. In contrast, the knowledge items were factual and dichotomous, and therefore not amenable to reliability testing using methods such as Cronbach’s alpha. In addition, the study did not capture the perspectives of other healthcare professionals, such as nurses. Finally, the possibility of response bias should be acknowledged, as participants may have provided socially desirable answers or overestimated their actual knowledge and practices related to MedRec.

Despite these limitations, the study has several strengths. It is the first to comprehensively assess the KAP of pharmacists and physicians regarding MedRec in Egypt, targeting the key professional groups responsible for its implementation. The study provides up-to-date, context-specific data that can inform national policies and educational strategies, and its findings are discussed within a robust regional and international framework. Although this study focused on Egyptian healthcare professionals, its implications extend to other LMICs with similar health system structures. In many such settings, the absence of clear institutional guidelines and limited training opportunities constrain MedRec implementation. Our findings highlight the importance of embedding MedRec training into undergraduate curricula, providing structured in-service education, and developing multidisciplinary policies that delineate responsibilities according to each profession’s strengths.

## Conclusion

In conclusion, while pharmacists and physicians in Egypt perceive themselves as central to the MedRec process, notable differences exist in their knowledge and practices. These gaps, particularly among physicians, highlight areas for targeted improvement. Addressing knowledge deficiencies is critical, as inadequate understanding of MedRec can contribute to medication errors, fragmented care, and potential patient harm. To strengthen implementation, structured workplace training programs targeting physicians should focus on enhancing familiarity with MedRec, while capacity-building initiatives for pharmacists should emphasize patient interviewing and documentation skills. Embedding MedRec training into undergraduate curricula and clearly defining roles within institutional policies will further support consistent practice across professions.

## Supplementary Information

Supplementary Material 1

Supplementary Material 2

## Data Availability

All data supporting the results reported in the manuscript are available upon request from the authors.

## Abbreviations

MedRec: Medication Reconciliation
NPSG: National Patient Safety Goals
BPMH: Best Possible Medication History
BPMDP: Best Possible Medication Discharge Plan
WHO: World Health Organization
SOP: Standard Operating Protocol
LMICs: Low- and Middle-Income Countries
ICU: Intensive Care Unit
KAP: Knowledge, Attitudes, and Practices
STROBE: Strengthening the Reporting of Observational Studies in Epidemiology
SPSS®: Statistical Package for Social Sciences
OR: Odds Ratio
CI: Confidence Interval
PhD: Doctor of Philosophy
MD: Doctor of Medicine
vs: Versus
