# Supplementary material for: Medication reconciliation in clinical practice: a survey of knowledge, attitude, and practices among Egyptian healthcare providers
Source: BMC Med Educ. 2025 Dec 19;26:130. doi: 10.1186/s12909-025-08285-2 (PMC12831293; doi:10.1186/s12909-025-08285-2)
Supplement: Supplementary file 1 — Supplementary Material 1 [file 12909_2025_8285_MOESM1_ESM.docx]

***Electronic Supplementary Materials***

- ***Submitted to***: BMC Medical Education Journal
- ***Title of the Article:*** “Medication Reconciliation in Clinical Practice: A Survey of Knowledge, Attitude, and Practices Among Egyptian Healthcare Providers”
- ***Authors and Affiliations:***
- ***Aya M. AbdelMagid***, Lecturer of Clinical Pharmacy, Cairo University
- ***Nirmeen A. Sabry***, Professor of Clinical Pharmacy, Cairo University
- ***Ahmed Abuelhana***, Lecturer of Clinical Pharmacy, School of Pharmacy & Pharmaceutical Sciences, Ulster University
- ***Aaron Courtenay***, Senior Lecturer of Clinical Pharmacy, School of Pharmacy & Pharmaceutical Sciences, Ulster University
- ***Amani M. Ali***, Lecturer of Clinical Pharmacy, Cairo University
- **Corresponding Author:**
- Aya M. Abdel Magid; address: Faculty of Pharmacy, Cairo University, Kasr El-Aini St., P.O. Box: 11562, Cairo, Egypt; telephone: +0201061115224.
- E-mail: [aya.abdelmagid@pharma.cu.edu.eg](mailto:aya.abdelmagid@pharma.cu.edu.eg), ORCID: 0000-0001-6175-1690

***Table S1: Demographics of Respondents who Answered all Questionnaire Questions***

| **Parameter** | **All  (N = 136)** | **Pharmacist**  **N = 88** | **Physician**  **N = 48** | **P value** |
| --- | --- | --- | --- | --- |
| **Gender** | | | | |
| Female | 88 (64.7) | 67 (76.1) | 21 (43.8) | **0.001*** |
| **Age (Years)** | | | | |
| 23-30 years | 45 (33.1) | 35 (39.8) | 10 (20.8) | **<0.001*** |
| 31-40 years | 46 (33.8) | 37 (42) | 9 (18.8) |  |
| 41-50 years | 32 (23.5) | 15 (17) | 17 (35.4) |  |
| 51-60 years | 11 (8.1) | 1 (1.1) | 10 (20.8) ^$^ |  |
| 61 or above | 2 (1.5) | 0 (0) | 2 (4.2) |  |
| **Highest Academic Degree** | | | | |
| Bachelor’s degree | 38 (27.9) | 34 (38.6) | 4 (8.3) ^$^ | **<0.001*** |
| Master’s degree | 48 (35.3) | 28 (31.8) | 20 (41.7) |  |
| Post-graduate diploma | 14 (10.3) | 14 (15.9) | 0 (0) ^$^ |  |
| PhD Degree | 6 (4.4) | 4 (4.5) | 2 (4.2) |  |
| MD Degree | 19 (14) | 0 (0) | 19 (39.6) ^$^ |  |
| Other | 11 (8.1) | 8 (9.1) | 3 (6.3) |  |
| **Years of Experience** | | | | |
| < 2 years | 10 (7.4) | 8 (9.1) | 2 (4.2) | 0.094 |
| 2 - 5 years | 33 (24.3) | 23 (26.1) | 10 (20.8) |  |
| 6 - 10 years | 27 (19.9) | 21 (23.9) | 6 (12.5) |  |
| > 10 years | 66 (48.5) | 36 (40.9) | 30 (62.5) |  |
| **Average Working Hours per Day** | | | | |
| < 8 hours | 30 (22.1) | 26 (29.5) | 4 (8.3) ^$^ | **0.004*** |
| ≥8 hours | 106 (77.9) | 62 (70.5) | 44 (91.7) |  |
| **Number of Patients Dealt with During a Typical Working Day** | | | | |
| None | 12 (8.8) | 11 (12.5) | 1 (2.1) | 0.079 |
| 1–10 | 29 (21.3) | 20 (22.7) | 9 (18.8) |  |
| 11–20 | 50 (36.8) | 26 (29.5) | 24 (50) |  |
| 21–30 | 16 (11.8) | 10 (11.4) | 6 (12.5) |  |
| > 30 | 29 (21.3) | 21 (23.9) | 8 (16.7) |  |
| **Working in a Hospital** | | | | |
| Yes | 136 (100) | 88 (100) | 48 (100) | **NA** |
| **Healthcare Institution Type** | | | | |
| Public | 92 (67.6) | 55 (62.5) | 37 (77.1) | 0.179 |
| Private | 37 (27.2) | 27 (30.7) | 10 (20.8) |  |
| Military | 7 (5.1) | 6 (6.8) | 1 (2.1) |  |
| Categorical data as Number (Percentages).  *: Level of significance P < 0.05, Chi Square (two -sided)  ^$^ Significant values of adjusted standardized residuals (absolute value > ±1.96 indicate statistical significance at p < 0.05)  Abbreviations: MD: Doctor of Medicine, PhD: Doctor of Philosophy | | | | |

***Table S2: Demographics of Respondents who Answered all Questionnaire Questions versus who did not Answer Practice Section Questions***

| **Parameter** | **Practice Only** | **No Practice** | **P-value** | **Practice Only** | **No Practice** | **P-value** | **Practice Only** | **No Practice** | **P-value** |
| --- | --- | --- | --- | --- | --- | --- | --- | --- | --- |
|  | **All  (N = 136)** | **All  (N = 136)** |  | **Pharmacist**  **N = 88** | **Pharmacist**  **N = 94** |  | **Physician**  **N = 48** | **Physician**  **N = 42** |  |
| **Gender** | | | | | | | | | |
| Female | 88 (64.7) | 106 (77.9) | **0.016*** | 67 (76.1) | 74 (78.7) | 0.676 | 21 (43.8) | **32 (76.2)** | **0.002*** |
| **Age (Years)** | | | | | | | | | |
| 23-30 years | 45 (33.1) | 45 (33.1) | 0.810 | 35 (39.8) | 33 (35.1) | 0.656 | 10 (20.8) | 12 (28.6) | 0.758 |
| 31-40 years | 46 (33.8) | 53 (39) |  | 37 (42) | 43 (45.7) |  | 9 (18.8) | 10 (23.8) |  |
| 41-50 years | 32 (23.5) | 27 (19.9) |  | 15 (17) | 17 (18.1) |  | 17 (35.4) | 10 (23.8) |  |
| 51-60 years | 11 (8.1) | 8 (5.9) |  | 1 (1.1) | 0 (0) |  | 10 (20.8) | 8 (19) |  |
| 61 or above | 2 (1.5) | 3 (2.2) |  | 0 (0) | 1 (1.1) |  | 2 (4.2) | 2 (4.8) |  |
| **Highest Academic Degree** | | | | | | | | | |
| Bachelor’s degree | 38 (27.9) | 46 (33.8) | **< 0.001*** | 34 (38.6) | 34 (36.2) | **<0.001*** | 4 (8.3) | 12 (28.6) | 0.097 |
| Master’s degree | 48 (35.3) | 32 (23.5) ^$^ |  | 28 (31.8) | 20 (21.3) |  | 20 (41.7) | 12 (28.6) |  |
| Post-graduate diploma | 14 (10.3) | 9 (6.6) |  | 14 (15.9) | 8 (8.5) |  | 0 (0) | 1 (2.4) |  |
| PhD Degree | 6 (4.4) | 31 (22.8) ^$^ |  | 4 (4.5) | 31 (33) ^$^ |  | 2 (4.2) | 0 (0) |  |
| MD Degree | 19 (14) | 15 (11) |  | 0 (0) | 0 (0) |  | 19 (39.6) | 15 (35.7) |  |
| Other | 11 (8.1) | 3 (2.2) ^$^ |  | 8 (9.1) | 1 (1.1) ^$^ |  | 3 (6.3) | 2 (4.8) |  |
| **Years of Experience** | | | | | | | | | |
| < 2 years | 10 (7.4) | 9 (6.6) | 0.992 | 8 (9.1) | 4 (4.3) | 0.502 | 2 (4.2) | 5 (11.9) | 0.529 |
| 2 - 5 years | 33 (24.3) | 33 (24.3) |  | 23 (26.1) | 23 (24.5) |  | 10 (20.8) | 10 (23.8) |  |
| 6 - 10 years | 27 (19.9) | 26 (19.1) |  | 21 (23.9) | 21 (22.3) |  | 6 (12.5) | 5 (11.9) |  |
| > 10 years | 66 (48.5) | 68 (50) |  | 36 (40.9) | 46 (48.9) |  | 30 (62.5) | 22 (52.4) |  |
| **Average Working Hours per Day** | | | | | | | | | |
| < 8 hours | 30 (22.1) | 55 (40.4) ^$^ | **0.001*** | 26 (29.5) | 43 (45.7) ^$^ | **0.024*** | 4 (8.3) | 12 (28.6) ^$^ | **0.012*** |
| ≥8 hours | 106 (77.9) | 81 (59. 6) ^$^ |  | 62 (70.5) | 51 (54.3) ^$^ |  | 44 (91.7) | 30 (71.4) ^$^ |  |
| **Number of Patients Dealt with During a Typical Working Day** | | | | | | | | | |
| None | 12 (8.8) | 65 (47.8) ^$^ | **<0.001*** | 11 (12.5) | 64 (68.1) ^$^ | **<0.001*** | 1 (2.1) | 1 (2.4) | 0.415 |
| 1–10 | 29 (21.3) | 26 (19.1) |  | 20 (22.7) | 16 (17) |  | 9 (18.8) | 10 (23.8) |  |
| 11–20 | 50 (36.8) | 20 (14.7) ^$^ |  | 26 (29.5) | 5 (5.3) ^$^ |  | 24 (50) | 15 (35.7) |  |
| 21–30 | 16 (11.8) | 7 (5.1) ^$^ |  | 10 (11.4) | 4 (4.3) |  | 6 (12.5) | 3 (7.1) |  |
| > 30 | 29 (21.3) | 18 (13.2) |  | 21 (23.9) | 5 (5.3) ^$^ |  | 8 (16.7) | 13 (31) |  |
| **Working in a Hospital** | | | | | | | | | |
| Yes | 136 (100) | 49 (36) | **<0.001*** | 88 (100) | 10 (10.6) | **<0.001*** | 48 (100) | **3 (7.1)** | **0.06*** |
| **Healthcare Institution Type** | | | | | | | | | |
| Public | 92 (67.6) | 34(69.4) | 0.155 | 55 (62.5) | 7 (70) | **0.015*** | 37 (77.1) | 27 (69.2) | 0.427 |
| Private | 37 (27.2) | 9 (18.4) |  | 27 (30.7) | 0 (0) ^$^ |  | 10 (20.8) | 9 (23.1) |  |
| Military | 7 (5.1) | 6 (12.2) |  | 6 (6.8) | 3 (30) ^$^ |  | 1 (2.1) | 3 (7.7) |  |
| Categorical data as Number (Percentages).  *: Level of significance P < 0.05, Chi Square (two -sided)  ^$^ Significant values of adjusted standardized residuals (absolute value > ±1.96 indicate statistical significance at p < 0.05)  Abbreviations: MD: Doctor of Medicine, PhD: Doctor of Philosophy | | | | | | | | | |
